# Efficacy of rifaximin against covert hepatic encephalopathy and hyperammonemia in Japanese patients

**DOI:** 10.1371/journal.pone.0270786

**Published:** 2022-07-01

**Authors:** Masato Nakai, Goki Suda, Koji Ogawa, Sonoe Yoshida, Shunichi Hosoda, Akinori Kubo, Yoshimasa Tokuchi, Takashi Kitagataya, Ren Yamada, Taku Shigesawa, Masatsugu Ohara, Takuya Sho, Kenichi Morikawa, Naoya Sakamoto

**Affiliations:** Department of Gastroenterology and Hepatology, Hokkaido University Graduate School of Medicine, Sapporo, Japan; University of Frankfurt: Goethe-Universitat Frankfurt am Main, GERMANY

## Abstract

Covert hepatic encephalopathy (CHE) impairs patient quality of life and occurs in approximately 30% of liver cirrhosis (LC) cases. Japanese clinical practice guidelines recommend rifaximin to treat overt HE (OHE). However, the usefulness of rifaximin against CHE is not thoroughly investigated in Japanese patients. We aimed to investigate the efficacy of rifaximin against hyperammonemia and CHE in Japan. We observed 102 patients with HE showing hyperammonemia secondary to LC and examined various biochemical and behavioral parameters following rifaximin treatment. CHE was diagnosed when the patients exhibited two or more abnormal neuropsychological test (NPT) scores but did not indicate OHE symptoms. In the 102 cases, a significant therapeutic effect of rifaximin on hyperammonemia was observed from 2 to 48 weeks after starting treatment. Excluding 10 patients diagnosed with OHE upon starting rifaximin treatment, 12 of the 92 remaining patients (11.8%) transitioned to OHE within 1 year. The 1 year cumulative OHE transition rate was 14.5%. Among the 24 patients with CHE diagnosed by the NPT for whom NPT results could be evaluated at 4 and 12 weeks after starting treatment, 10 (41.6%) had recovered from CHE at 12 weeks. When the factors contributing to recovery from CHE were examined by multivariate analysis, an ammonia level <129 μg/dL was a significant factor. Rifaximin was thus significantly effective against both hyperammonemia and CHE in Japanese patients.

## Introduction

Hepatic encephalopathy (HE) is a neuropsychiatric syndrome caused by chronic liver conditions including cirrhosis, acute liver failure, and portosystemic shunts. Symptoms such as personality changes and sleep rhythm disorders may occur early. However, asterixis and impaired consciousness can manifest in the advanced stage, leading to coma and death [1, 2]. The West Haven Criteria (WHC) [1] and International Society of Hepatic Encephalopathy (ISHEN) [3] classification systems are commonly used to classify HE progression. In the Liver Cirrhosis Clinical Practice Guidelines [4] published in Japan in 2020, HE is described as overt (OHE) or covert (CHE) based on the ISHEN classification. Furthermore, CHE is classified as either first-degree or minimal hepatic encephalopathy (MHE) according to the WHC; it can be diagnosed in the absence of conspicuous symptoms, such as asterixis.

CHE is often not appropriately diagnosed in the clinical setting. MHE reportedly occurs in approximately 30% of the cases of liver cirrhosis [5]. Furthermore, since the transition from MHE to OHE is observed at a certain rate and can cause the deterioration of automobile driving skills or quality of life [6], the appropriate and timely diagnosis of CHE is considered essential. Various quantitative methods for diagnosing CHE have been reported. Along with the psychometric hepatic encephalopathy score [1] and the animal naming test [7], neuropsychological tests (NPT) [8] comprise a widely accepted quantitative method, especially in Japan. An NPT application that can be inspected on a touch screen tablet has been made available by The Japan Society of Hepatology. In this application, eight tests [number connection test-A and -B, a figure position test, a digit symbol test, a block design test, and Reaction test-A, B, and C] could be performed in the previous version; however, six tests (Stroop test instead of Reaction test -A, B and C) can be performed in the latest version.

In addition to diagnostic tools, treatment options for HE are crucial. Synthetic disaccharide laxatives, such as lactulose, are standard treatments for OHE; however, the use of laxatives is often difficult because of their poor palatability and side effects, such as diarrhea. Rifaximin was first developed in Italy in 1985 and has been available for OHE treatment in Europe and the United States since 2010 [9, 10]. In Japan, this drug has been approved for HE treatment since September 2016 [11]. In the Liver Cirrhosis Clinical Practice Guidelines [4], rifaximin is recommended as a second-line HE treatment following synthetic disaccharide laxative use. However, this drug is a poorly absorbed antibiotic that directly suppresses ammonia production by the gut microbiota. It has been widely used in Europe and the United States, but its therapeutic effects have not been investigated in Japan. Particularly, its effectiveness against CHE has not yet been sufficiently evaluated. In this study, we investigated the efficacy of rifaximin against hyperammonemia and CHE diagnosed using NPT in a Japanese population.

## Materials and methods

### Study design and participants

This was a prospective observational cohort study. We enrolled patients with liver cirrhosis (LC) who were diagnosed with HE by an attending physician and prescribed rifaximin at Hokkaido University Hospital between January 2018 and July 2021. LC was diagnosed by liver biopsy, Fibroscan data, radiologic findings, such as computed tomography or magnetic resonance imaging, and laboratory data, according to a previous report [12]. The inclusion criteria were as follows: age ≥ 20 years, blood ammonia level ≥ 70 μg/dL, and HE diagnosis as determined by the attending physician. HE grading was diagnosed based on an interview and medical examination. First-degree encephalopathy was judged extensively on the basis of changes in behavioral patterns and the mental status during daily life activities using the WHC. The exclusion criteria were the presence of tuberculosis (pulmonary and other forms); pregnancy or lactation; diagnosis of pseudomembranous enteritis and other psychiatric disorders, such as apparent dementia; acute liver failure; and a judgment of ineligibility by the attending physician. Written informed consent was obtained from all participants.

**Fig 1:**
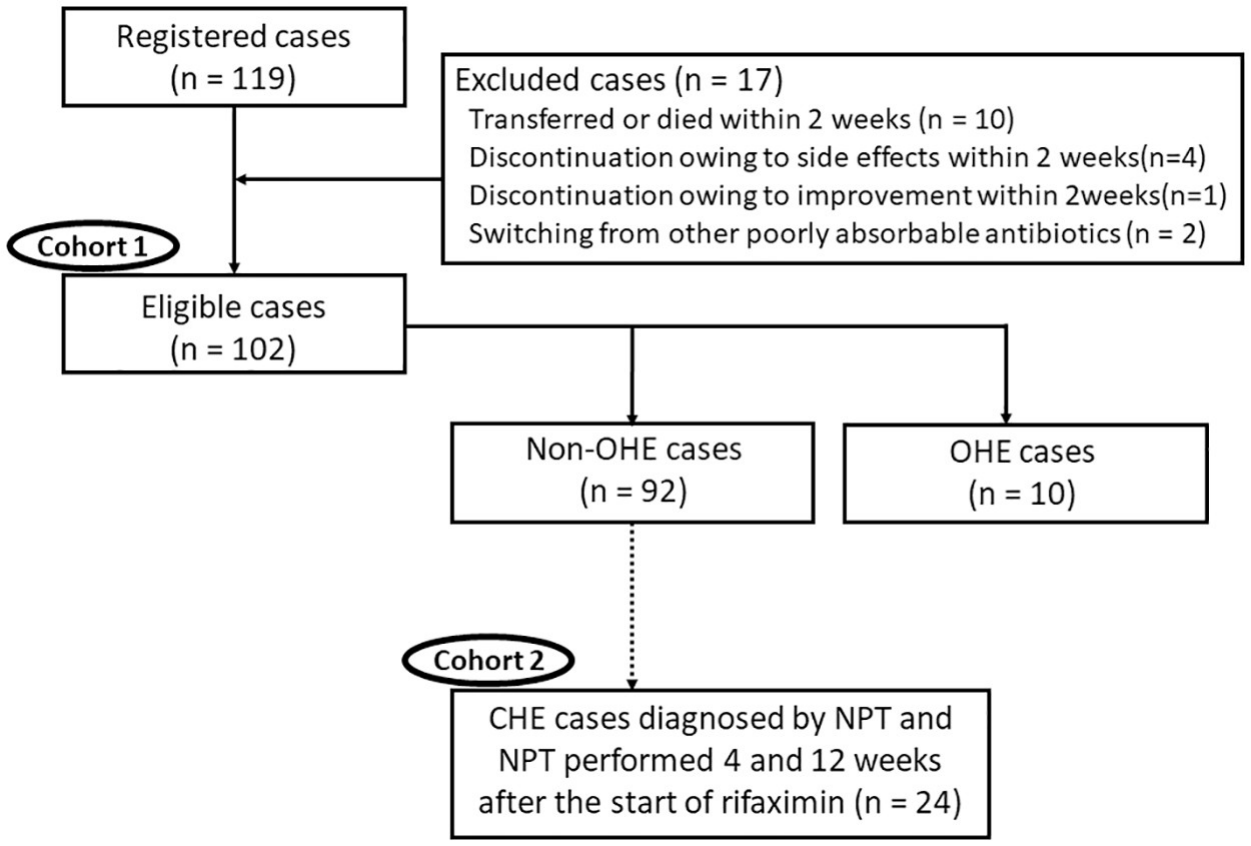
Patient flow chart. Flowchart indicating patient classification. NPT, neuropsychological tests; OHE, overt hepatic encephalopathy; CHE, covert hepatic encephalopathy.

As shown in Fig 1, this study was conducted based on two cohorts. In cohort 1, we investigated the therapeutic effect of rifaximin on hyperammonemia in all eligible cases. Additionally, the rates of transition to OHE in non-OHE cases were analyzed. In cohort 2, we investigated the therapeutic effect of rifaximin on CHE in patients who were diagnosed with CHE by NPT and agreed to NPT at 4 and 12 weeks after treatment with rifaximin.

### Patient follow-up and additional treatment

The registered patients underwent blood tests before starting rifaximin treatment. Clinical symptoms, including asterixis, were evaluated. OHE was diagnosed according to the ISHEN criteria [3] and was defined by symptoms such as asterixis and disorientation. Background clinical data and the history of OHE were determined from interviews and clinical records. In cases where computed tomography data were available before starting treatment, muscle volume loss was determined by the simple method of examining the iliopsoas muscle at the third lumbar vertebra level using the Japanese Society of Hepatology criteria [13]. Additionally, the computed tomography data were used to determine the presence of a portosystemic shunt (≥5 mm), and the maximum diameter was measured. In cohort 1, blood samples were tested for ammonia levels and other clinical parameters, and an evaluation of the presence of OHE was conducted at 2, 4, 8, 12, 24, 36, and 48 weeks after starting treatment. Ammonia levels following the treatment were evaluated until rifaximin was discontinued, additional treatment for HE was initiated, or the dose of concomitant medications were increased to eliminate the effects of other treatments. Specifically, patients who received additional treatment or discontinued rifaximin were analyzed as censored. Additional treatment included the administration of synthetic disaccharides, other nonabsorbable antibiotics, branched-chain amino acids (BCAAs), zinc, and L-carnitine, as well as clinical procedures such as interventional shunt occlusion (such as balloon-occluded transfemoral obliteration) and partial splenic embolization. No additional nutritional counseling was provided for hyperammonemia during the observation period.

### NPT and CHE diagnosis

In cohort 2, NPT were performed before treatment and at 4 and 12 weeks after treatment for patients who consented to further evaluation. The test was performed on a touch screen tablet using an application [8] developed by the Japanese Society of Hepatology. Medical staff assisted with the operation to eliminate the bias caused by user error. The cut-off score of each test was determined using the reference value for each age group, according to a previous report [8]. Among the tests included in the NPT, four tests (number connection tests A and B, digit symbol test, and block design test) were used to evaluate CHE. According to previous reports [1, 5, 14], CHE was diagnosed when the results of two or more of the NPT were abnormal and when the patient did not present symptoms that are characteristic of OHE, such as asterixis or disorientation. The proportion of patients with CHE before treatment and the proportion of patients who recovered from CHE after rifaximin administration were examined. Patients receiving additional treatment after the start of rifaximin were defined as not showing recovery from CHE, even if the NPT score improved over time.

### Ethical considerations

The study protocol was approved by the institutional ethics committee of Hokkaido University (Trial Number: 017–0313) and conformed to the ethical guidelines of the Declaration of Helsinki. Written informed consent was obtained from all patients. The study was registered in the UMIN Clinical Trials Registry (UMIN000044146).

### Statistical analysis

The Mann–Whitney U test was used to compare the data between two groups and analyze continuous variables. Fisher’s exact test was used for the univariate analysis of categorical independent variables. Logistic regression and multivariate analyses were used for categorical independent variables. Receiver operating characteristic (ROC) curves were drawn to determine the optimal cut-off values for multivariate analysis. For the time course of a variable, the Wilcoxon signed-rank test was used for testing the significant difference between the two points. The rate of transition to OHE was analyzed using Gray’s test. All statistical analyses were performed using EZR software [15].

## Results

### Patient characteristics

Fig 1 shows the patient classification flowchart. Rifaximin administration was started for 119 patients with LC who exhibited hyperammonemia during the observation period. Within 2 weeks, 10 of these patients had died or were transferred to different hospitals. Treatment was discontinued for four patients within 2 weeks owing to side effects, as determined by the attending physician as follows: one developed constipation (grade 2), two developed a rash (grade 2), and one developed fatigue (grade 2). All patients recovered, and no other physical disorders or ailments were observed. Treatment of one patient was discontinued within 2 weeks following an assessment by the attending physician, who concluded that HE had improved in the patient. Additionally, two patients switched to rifaximin from other nonabsorbable antibiotics. Therefore, these 17 patients were excluded from further evaluation. We analyzed changes in the ammonia levels of the remaining 102 patients who received continuous rifaximin for 2 weeks or longer as cohort 1. Table 1 presents patient characteristics. Rifaximin was administered three times daily. The starting dose was 1200 mg for 98 patients (96.1%). The median patient age was 67 years, with 61.8% of the study group comprising men. Regarding background liver diseases, 24 and 16 patients had hepatitis B virus and hepatitis C virus infections, respectively. The remaining 56 patients had non-B, non-C liver disease. Moreover, 28 patients (27.3%) exhibited advanced hepatocellular carcinoma based on the Milan criteria [16]. The Child–Pugh grade was A for eight cases, B for 69 cases, and C for 25 cases, whereas the median Model for End-Stage Liver Disease score was 11 points. Ten patients (9.8%) had OHE, as determined using the ISHEN criteria. Gastroesophageal varices were found in 67 patients (65.7%). There were 56 cases (54.9%) of a portosystemic shunt with a maximum diameter of ≥5 mm, and the median maximum diameter was 8 mm (<5–30 mm). The concomitant medications at the initiation of rifaximin treatment were BCAAs for 76 patients (74.5%), synthetic disaccharides for 73 (71.6%), L-carnitine for 45 (44.1%), and zinc for six (5.9%). The median medication period of rifaximin was 227 days.

**Table 1:** Baseline characteristics of patients (Cohort 1).

Number	102
Sex, male n (%)	63 (61.8)
Age (years) ^a^	67 (20–83)
Body mass index (kg/m^2^) ^a^	24.1 (16.8–40.9)
Etiology, n (%)	
Hepatitis B virus	24 (23.5)
Hepatitis C virus	16 (15.7)
Alcohol	17 (16.7)
Non-alcoholic steatohepatitis	23 (22.5)
Others or Unknown	22 (21.6)
HCC, up to the Milan criteria, n (%)	28 (27.5)
Child-Pugh grade (A/B/C)	8 / 69 / 25
MELD score (points) ^a^	11 (6–32)
Starting dose of rifaximin, 1200 mg n (%)	98 (96.1)
History of OHE, yes, n (%)	18 (17.6)
Muscle volume loss (JSH criteria), n (%)	47 (47.5)^b^
Concomitant medication, n (%)	
BCAA	76 (74.5)
Synthetic disaccharides	73 (71.6)
L-carnitine	45 (44.1)
Zn	6 (5.9)
Loop diuretics	43 (42.2)
PPI/PCAB	69 (67.6)
Gastroesophageal varices, yes n (%)	67 (65.7)
Portosystemic shunts >5 mm, yes n (%)	56 (54.9)
Maximum diameter of portosystemic shunt (mm) ^a^	8.0 (<5.0–30.0)
Diabetes, n (%)	40 (40.2)
Hypertension, n (%)	40 (40.2)
NH_3_ (μg/dL) ^a^	117 (71–277)
Albumin (g/dL) ^a^	3.2 (1.6–4.4)
Total bilirubin (mg/dL) ^a^	1.5 (0.2–21.0)
PT-INR ^a^	1.25 (0.98–2.10)
Serum creatinine (mg/dL) ^a^	0.71 (0.33–3.40)
CRP (mg/dL) ^a^	0.17 (0.02–6.58)
WBC (/mm^3^) ^a^	4450 (1700–9700)
NLR ^a^	2.67 (0.52–14.33) ^c^
BTR ^a^	3.45 (1.30–12.84) ^d^
Median medication period (days) ^a^	227 (21–1369)

^a^ Median (range)

^b^ The data included only 99 patients;

^c^ the data included only 88 patients;

^d^ the data included only 29 patients

HCC, hepatocellular carcinoma; MELD, The model for End-stage Liver Disease; HE, hepatic encephalopathy; OHE, overt hepatic encephalopathy; JSH, The Japan Society of Hepatology; BCAA, branched chain amino acid; PPI, proton pump inhibitor; PCAB, potassium-competitive acid blocker; NH_3_, ammonia; PT-INR, prothrombin time-international normalized ratio; CRP, C-reactive protein; WBC, white blood cell; NLR, neutrophil/lymphocyte ratio; BTR, branched chain amino acids/tyrosine molar ratio

### Ammonia level changes following rifaximin administration

**Fig 2:**
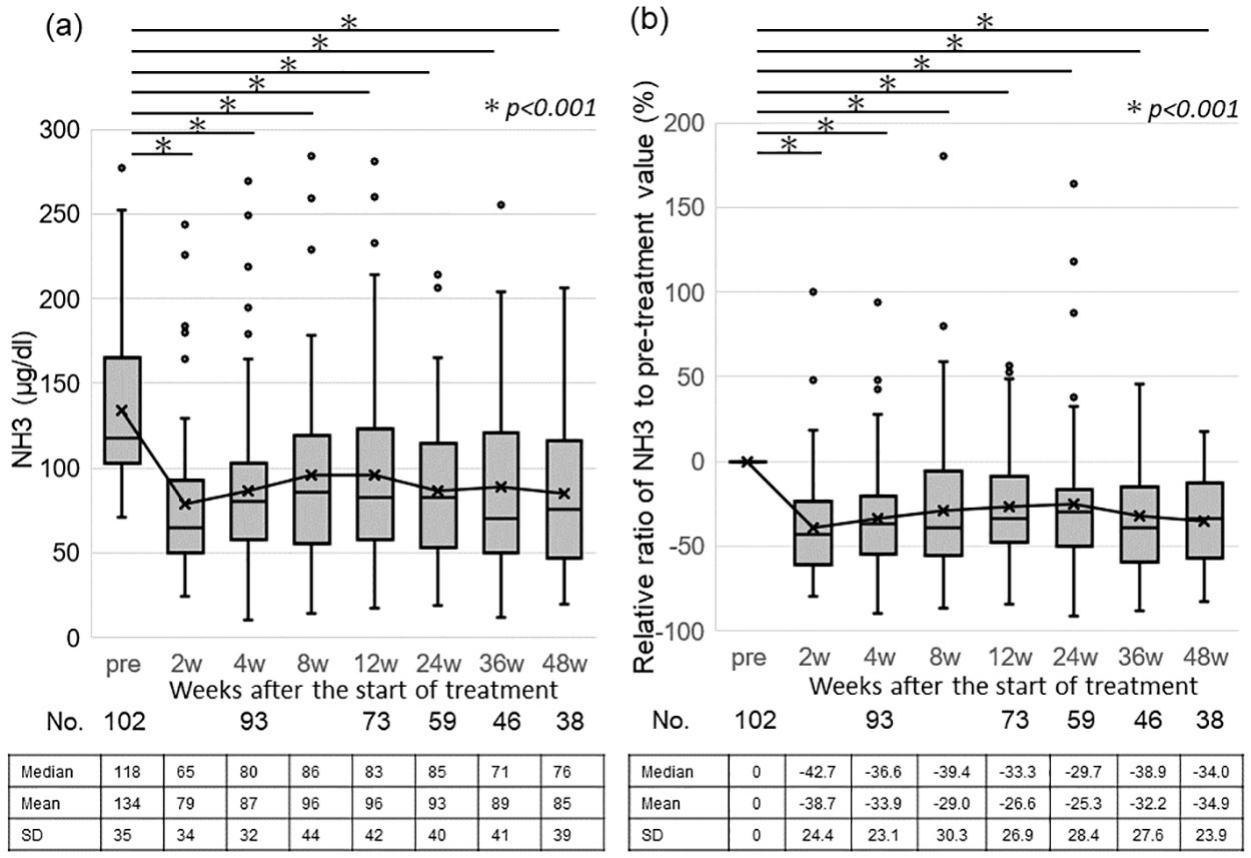
Effect of rifaximin treatment on hyperammonemia. (**a**) Changes in the blood ammonia levels in patients from pre-treatment to 48 weeks after starting treatment are shown on the Y-axis. (**b**) Changes in the relative ratio of the ammonia level measured during treatment to its pre-treatment value are shown on the Y-axis. In each graph, plots and data represent the mean ± standard deviation (SD).

Blood ammonia levels of the patients showed a significant decrease after rifaximin administration, from 118 μg/dL before treatment to 65 μg/dL (−42.7%; P < 0.01), 80 μg/dL (−36.6%; P < 0.01), 86 μg/dL (−39.4%; P < 0.01), 83 μg/dL (−33.3%; P < 0.01), 85 μg/dL (−29.7%; P < 0.01), and 76 μg/dL (−34.0%; P < 0.01) at 2, 4, 8, 12, 24, and 48 weeks, respectively. A significant decrease was observed at 48 weeks after starting treatment (Fig 2).

### Transition to OHE and HE recurrence

**Fig 3:**
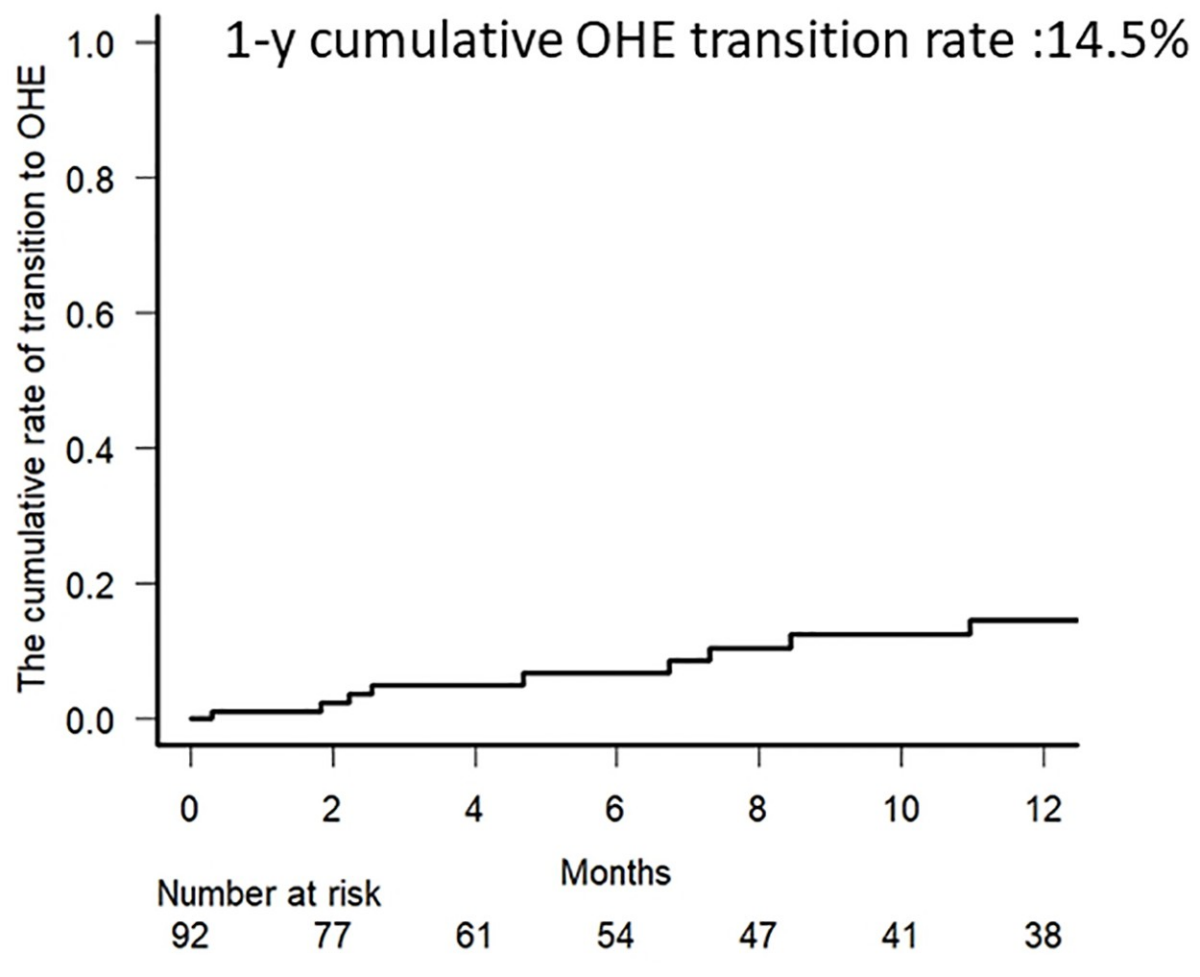
One-year cumulative transition rate to overt hepatic encephalopathy (OHE).

Of the 92 patients without OHE at the initiation of rifaximin treatment in cohort 1, 12 (11.8%) transitioned to OHE within 12 months of treatment, with a 1-year cumulative OHE transition rate of 14.5% (Fig 3). In 18 cases (19.6%), additional treatment for hyperammonemia was administered at the attending physician’s discretion, and a total of 30 patients (35.3%) required additional treatment for hyperammonemia or HE. The 1-year cumulative additional treatment rate was 29.4%.

### Therapeutic effect of rifaximin on CHE

**Fig 4:**
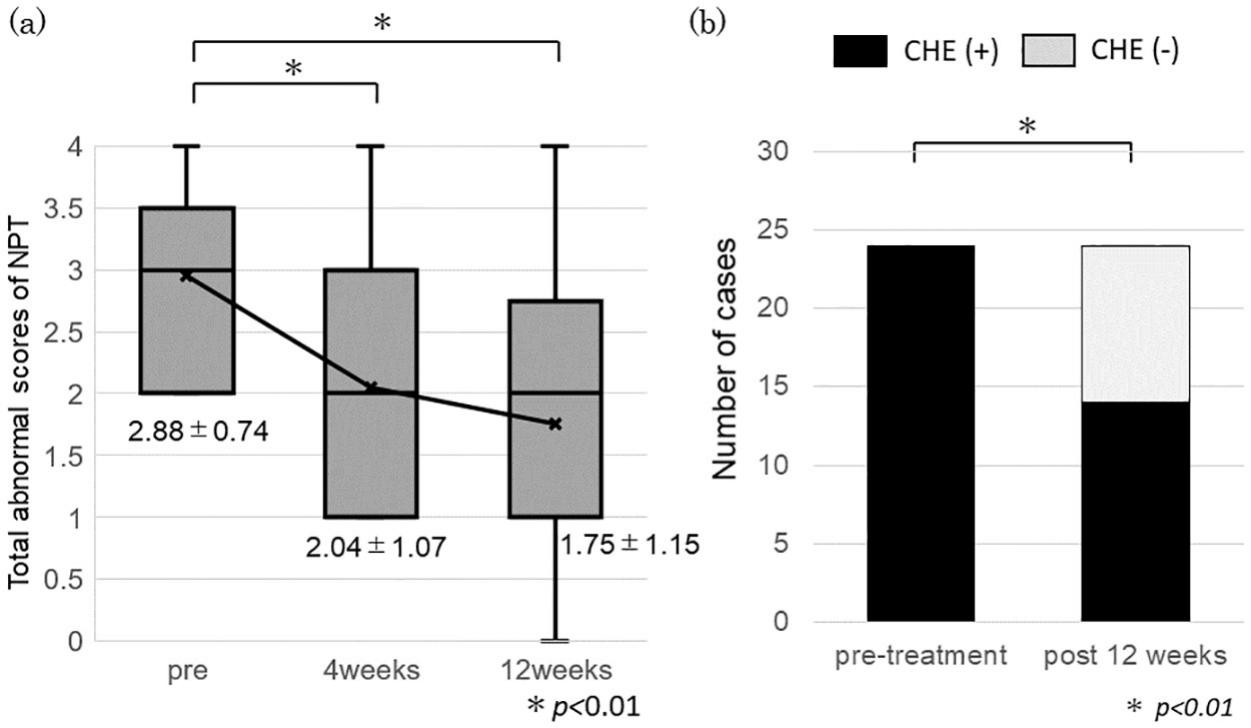
Effect of rifaximin in improving covert hepatic encephalopathy (CHE). (**a**) Total abnormal scores of neuropsychological tests (NPT) are shown on the Y-axis. Plots and data represent the mean ± SD. (**b**) Number of patients with CHE before and 12 weeks after treatment is shown on the Y-axis.

To investigate the effect of rifaximin on CHE, the results of the NPT before and after rifaximin administration were analyzed as cohort 2. Twenty-four patients diagnosed as having CHE by an NPT (before treatment) provided data for up to 12 weeks after starting treatment. Table 2 also shows the characteristics of these 24 patients. The average number of abnormal NPT scores was 2.9 before, 2.0 at 4 weeks after, and 1.8 at 12 weeks after treatment initiation. A significant decrease was observed at 4 and 12 weeks after treatment initiation (P < 0.01; Fig 4a). Ten patients (41.6%) did not meet the diagnostic criteria for CHE 3 months after treatment initiation, and the CHE complication rate was significantly reduced (P < 0.01; Fig 4b).

**Table 2:** Baseline characteristics and pre-treatment predictive factors for recovery from CHE diagnosed by NPT (Cohort 2).

Factors	All patients (n = 24)	No recovery from CHE (n = 14)	Recovery from CHE (n = 10)	Univariate p-values	Multivariate Cut-off OR (95%CI) *p*-values		
Age (years) ^a^	66 (20–80)	67 (44–80)	66 (20–75)	0.89			
Sex (male/female)	16 / 8	9 / 5	7 / 3	1.0			
Body mass index (kg/m^2^) ^a^	24.7 (17.8–33.9)	24.6 (17.8–32.8)	25.4 (18.6–33.9)	0.89			
Etiology (HBV/HCV/NBNC)	7 / 2/ 15	4 / 1 / 9	3 / 1 / 6	1.0			
History of OHE (yes/no)	2 / 22	2 / 12	0 / 10	0.50			
HCC (up to the Milan criteria; yes/no)	4 / 20	3 / 11	1 / 9	0.62			
Child-Pugh score ^a^	8 (6–11)	8 (6–11)	8 (6–9)	0.23			
MELD score ^a^	10 (7–15)	10 (7–15)	11 (9–15)	0.22			
Muscle volume loss (yes/no)	9 / 15	6 / 8	3 / 7	0.68			
Diabetes (yes/no)	9 / 15	7 / 7	2 / 8	0.21			
BCAA (yes/no)	16 / 8	10 / 4	6 / 4	0.68			
Synthetic disaccharides (yes/no)	15 / 9	6 / 8	9 / 1	**0.04**	Yes	11.4 (0.89–146.0)	0.07
L-Carnitine (yes/no)	7 / 17	3 / 11	4 / 6	0.40			
Zinc (yes/no)	1 / 23	1 / 13	0 / 10	1.0			
Loop diuretics (yes/no)	9 / 15	7 / 7	2 / 8	0.21			
PPI/PCAB (yes/no)	11 /13	8 / 6	3 / 7	0.24			
Gastroesophageal varices (yes/no)	15 / 9	7 / 7	8 / 2	0.21			
Portosystemic shunts (≥5 mm/<5 mm)	13 / 11	7 / 7	6 / 4	0.66			
**NH**_**3**_ **(μg/dL)** ^a^	123 (74–277)	**101 (74–175)**	**177 (111–277)**	**<0.01**	**129**	**9.5 (1.10–83.0)**	**0.04**
Albumin (g/dL) ^a^	3.5 (2.3–3.9)	3.4 (2.3–3.9)	3.5 (3.2–3.9)	0.22			
Total bilirubin (mg/dL) ^a^	1.6 (0.6–3.4)	1.6 (0.6–2.9)	1.6 (1.0–3.4)	0.53			
PT-INR ^a^	1.23 (1.03–1.67)	1.21 (1.03–1.62)	1.25 (1.13–1.67)	0.43			
Serum creatinine (mg/dL) ^a^	0.69 (0.44–1.17)	0.65 (0.44–0.98)	0.75 (0.52–1.17)	0.35			
CRP (mg/dL) ^a^	0.12 (0.02–1.18)	0.18 (0.02–1.18)	0.09 (0.02–0.54)	0.12			
WBC (/mm^3^) ^a^	3650 (1700–8400)	4650 (1700–8400)	3450 (1800–4500)	0.11			
NLR ^a^ ^b^	2.39 (0.68–7.09)	2.44 (1.18–7.09)	2.31 (0.68–6.42)	0.92			

^a^ Median (range);

^b^ The data included only 21 patients

CHE, covert hepatic encephalopathy; HBV, Hepatitis B virus; HCV, Hepatitis C virus; NBNC non-HBV non-HCV; OHE, overt hepatic encephalopathy; HCC, hepatocellular carcinoma; MELD, The model for End-stage Liver Disease; NH_3_, ammonia; PT-INR, prothrombin time-international normalized ratio; CRP, C-reactive protein; WBC, white blood cell; NLR, neutrophil/lymphocyte ratio

### Predictive factors for recovery from CHE after rifaximin treatment

**Fig 5:**
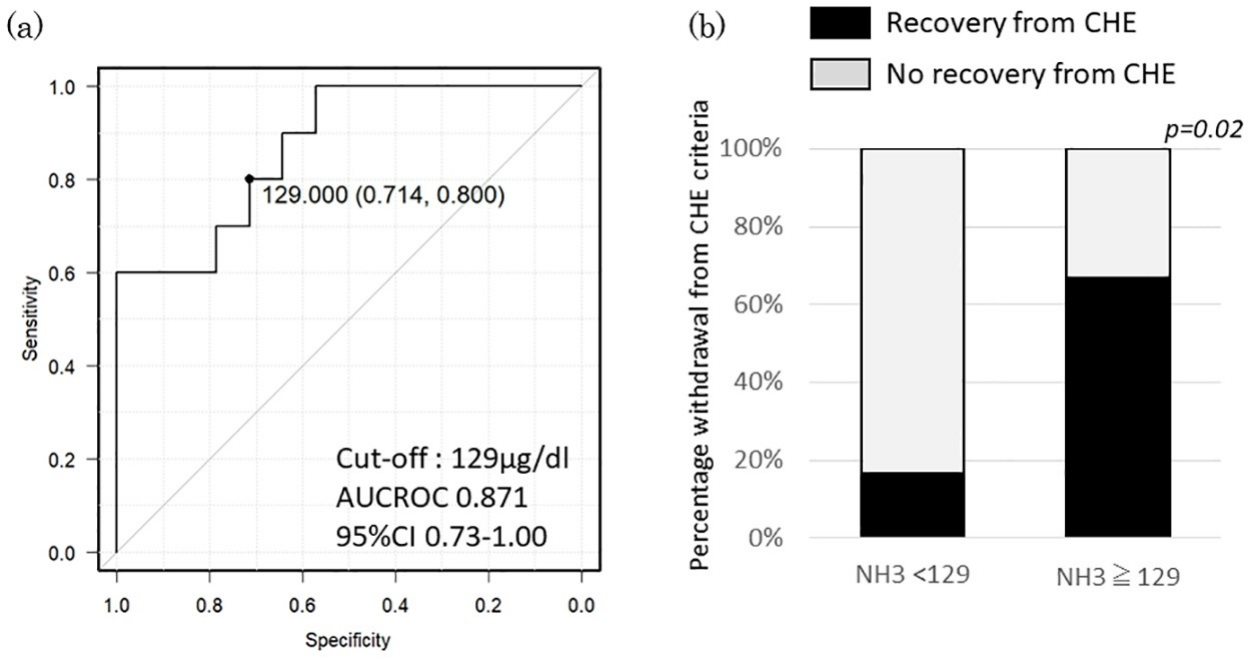
Ammonia levels before treatment with rifaximin and after recovery from covert hepatic encephalopathy (CHE). (**a**) Receiver operating characteristic curve showing the baseline ammonia level that is predictive of recovery from CHE at 12 weeks after rifaximin administration. (**b**) The Y-axis presents the percentage of cases showing recovery from CHE when cases were categorized according to the ammonia level (NH_3_ < 129 μg/dL and NH_3_ ≥ 129 μg/dL).

Next, we examined the factors involved in the recovery from CHE after rifaximin treatment. In the univariate analysis, high ammonia levels and the concomitant use of synthetic disaccharide laxatives were the predictive factors for recovery from CHE, as indicated by significant differences (P < 0.05). Using ROC curve analysis, we obtained a cut-off value of 129 μg/dL for the blood ammonia level (Fig 5a). In the multivariate analysis using these factors, an ammonia level greater than 129 μg/dL was extracted as a factor that significantly predicted recovery from CHE (P = 0.04, Table 2). The recovery rate from CHE was 16.6% (2/12) in the group with an ammonia level <129 μg/dL and 66.7% (8/12) for the group with an ammonia level ≥129 μg/dL. The CHE recovery rate was significantly higher in the latter group (P = 0.02; Fig 5b).

## Discussion

We observed the prospective therapeutic effect of rifaximin in Japanese patients with LC and hyperammonemia. The ammonia level decreased significantly for 48 weeks after starting treatment. Additionally, approximately 40% of the patients exhibiting CHE before treatment did not meet the diagnostic criteria for CHE 3 months after starting treatment. In particular, recovery from CHE was likely to occur in cases with high blood ammonia levels before beginning treatment.

Rifaximin has been used as a therapeutic agent for HE in Europe and the United States for many years, and there have been many reports of its therapeutic effect in patients in these countries. Rifaximin reportedly suppresses the recurrence of OHE and significantly reduces HE-related hospitalization compared with observations with a placebo [9]. In addition, rifaximin suppresses HE recurrence and increases the survival rate by reducing the gastroesophageal varix rupture rate, spontaneous bacterial peritonitis incidence, and hepatorenal syndrome incidence [17]. Therefore, rifaximin might help to effectively control LC complications other than HE. However, in Japan, rifaximin was approved quite recently; thus, data on the effectiveness of rifaximin in Japanese patients are still limited. Recently, Sezaki et al. reported a significant decrease in ammonia levels following rifaximin administration in Japanese patients with HE [18]. Kawaratani et al. [19] also reported the long-term efficacy of rifaximin in Japanese patients by analyzing ammonia levels in a multicenter study. Here, rifaximin administration significantly reduced blood ammonia levels (Fig 2). Therefore, rifaximin might be an effective treatment for OHE and hyperammonemia in Japanese patients.

CHE has been attracting attention in recent years because it reduces patient quality of life and is associated with poor work capacity [20, 21], poor driving ability [6, 22], and sleep disorders [23]. There are various reports on the prevalence of CHE and MHE, with MHE prevalence ranging between 21 and 85% and CHE prevalence ranging between 35.8 and 50.9% among LC patients, with varying frequencies, depending on the population [5, 14, 24–30]. According to reports from Japan, the prevalence of MHE is approximately 21% [14] or 30% [5].

Additionally, because CHE has been reported to transition to OHE at a consistent rate [24, 29, 31], it is important to treat it promptly. Although it is not recommended that all CHE cases be treated following Japanese and the American Association for the Study of Liver Diseases/European Association for the Study of the Liver clinical practice guidelines, additional treatment is proposed for high-risk patients, such as those with worsening background liver conditions. Miwa et al. reported a high transition rate from MHE to OHE in cases of zinc deficiency, high ammonia levels, high serum creatinine levels, and low platelet counts [32]. Since only patients receiving rifaximin were enrolled in this observational study, evaluating a transition to OHE in the absence of therapeutic intervention was not possible. However, since the rate of transition from CHE to OHE is reportedly high in patients with hyperammonemia [32], it is suggested that neuropsychological testing should be actively performed for at-risk patients to assess the presence of CHE, and in the case of a positive diagnosis, additional treatment should be considered.

Several reports have described the therapeutic effects of rifaximin on MHE [33–36]. In a randomized controlled trial comparing the effects of lactulose with those of rifaximin on MHE, recovery from MHE reportedly occurred in approximately 70% of the cases, with no significant difference observed between the effects of lactulose and those of rifaximin [34]. In Japan, it has been reported that number connection test-A and digit symbol test scores significantly improved 2 weeks after rifaximin administration in OHE cases [37]. However, to the best of our knowledge, the therapeutic effects of rifaximin on CHE/MHE in Japan have not been reported. We evaluated CHE using NPT and prospectively observed patients treated with rifaximin. Unlike the patients in the trial mentioned previously, more than 70% of the patients in this study were already prescribed other therapeutic agents (BCAAs and synthetic disaccharide laxatives) because BCAAs and synthetic disaccharide laxatives are recommended as standard treatments for HE and hyperammonemia, according to Japanese liver cirrhosis clinical practice guidelines [4]. However, there are a certain number of cases of intolerance owing to the problem of sweetness or diarrhea. Almost all patients who were unable to take synthetic disaccharide laxatives had these problems in this study. Because the concomitant rate of synthetic disaccharide laxative (such as lactulose) use in this study was similar to that in past clinical reports in Japan [18, 19], our study is considered to reflect actual clinical practice. Even in cases wherein other therapeutic agents had already been administrated, rifaximin addition effectively ameliorated CHE in approximately 45% of all cases. Therefore, rifaximin administration appears to be a promising therapeutic agent for LC with CHE even when BCAAs and synthetic disaccharide laxatives have already been administered.

We also examined the characteristics of patients that are likely to show recovery from CHE. A high blood ammonia level before treatment was identified as a factor that significantly contributed to the recovery from CHE. Because high ammonia levels affect cognitive function [38, 39] and many CHE diagnostic tools, such as NPT, measure cognition, higher baseline ammonia levels associated with CHE recovery likely resulted in improved NPT scores. Various diagnostic methods for CHE, such as NPT, are greatly affected by cognitive function, which was considered the reason for the influence of high ammonia levels on CHE recovery. The results of these diagnostic methods could be modified for patients with other potential psychiatric disorders and dementia. Here, patients with apparent clinical symptoms of psychiatric disorders, dementia, and pre-existing diseases were excluded; however, accurate diagnosis by imaging tests, such as magnetic resonance imaging of the brain, could not be performed. It is challenging to diagnose potential cases of dementia and psychiatric disorders; therefore, we could have included CHE cases and potential cases of dementia and psychiatric disorders. Moreover, the therapeutic effect on CHE might have been greater for those without these complications. Blood ammonia levels are relatively low in most cases of dementia and psychiatric disorders, and thus, the therapeutic effect of rifaximin might have been low.

Hyperammonemia is deeply involved in the development of HE [40]. However, it has also been reported that ammonia levels do not correlate linearly with the severity of HE [41]. In recent years, it has been reported that there are various mechanisms other than ammonia, such as inflammation, oxidative stress, increased bile acids, and lactate, that play a role in the onset of HE [42]. Therefore, it is possible that the effect of rifaximin is poor for CHE patients for whom the ammonia level is relatively low. As CHE cases with high ammonia levels can be expected to benefit from rifaximin treatment, additional treatment with rifaximin may suppress the transition to OHE and improve the quality of life of eligible patients.

Nonetheless, this study had some limitations. First, this was an observational study involving a single hospital and a small number of patients. Only 24 patients could be evaluated for CHE recovery using NPT. Second, CHE was evaluated only by NPT and was not confirmed by other evaluation methods. It would be advantageous to assess the psychometric hepatic encephalopathy score and perform the animal naming test and/or Stroop test. Third, dementia and other psychiatric disorders were excluded based on clinical symptoms, but strict diagnoses using imaging tests were not performed. The drugs associated with these disorders could not be examined. Fourth, the addition of or increase in the drugs affecting HE indirectly after the initiation of rifaximin was not examined. Finally, there were no restrictions on the co-administration of other drugs before or while starting rifaximin treatment, as this was an observational study. Therefore, our findings may not purely reflect the effectiveness of rifaximin. These limitations are expected to be addressed in future interventional studies using several CHE evaluation methods with a large number of cases. In conclusion, rifaximin had a positive effect on not only hyperammonemia but also CHE and therefore may be a promising therapeutic option.

## References

[1] Ferenci P, Lockwood A, Mullen K, Tarter R, Weissenborn K, Blei AT. Hepatic encephalopathy—definition, nomenclature, diagnosis, and quantification: final report of the working party at the 11th World Congresses of Gastroenterology, Vienna, 1998. Hepatology. 2002;35(3):716–21. doi: 10.1053/jhep.2002.31250 . 11870389

[2] Vilstrup H, Amodio P, Bajaj J, Cordoba J, Ferenci P, Mullen KD, et al. Hepatic encephalopathy in chronic liver disease: 2014 Practice Guideline by the American Association for the Study of Liver Diseases and the European Association for the Study of the Liver. Hepatology. 2014;60(2):715–35. doi: 10.1002/hep.27210 . 25042402

[3] Bajaj JS, Cordoba J, Mullen KD, Amodio P, Shawcross DL, Butterworth RF, et al. Review article: the design of clinical trials in hepatic encephalopathy—an International Society for Hepatic Encephalopathy and Nitrogen Metabolism (ISHEN) consensus statement. Aliment Pharmacol Ther. 2011;33(7):739–47. doi: 10.1111/j.1365-2036.2011.04590.x 21306407 PMC3971432

[4] Yoshiji H, Nagoshi S, Akahane T, Asaoka Y, Ueno Y, Ogawa K, et al. Evidence-based clinical practice guidelines for liver cirrhosis 2020. J Gastroenterol. 2021;56(7):593–619. doi: 10.1007/s00535-021-01788-x 34231046 PMC8280040

[5] Kato A, Tanaka H, Kawaguchi T, Kanazawa H, Iwasa M, Sakaida I, et al. Nutritional management contributes to improvement in minimal hepatic encephalopathy and quality of life in patients with liver cirrhosis: A preliminary, prospective, open-label study. Hepatol Res. 2013;43(5):452–8. doi: 10.1111/j.1872-034X.2012.01092.x . 22994429

[6] Bajaj JS, Heuman DM, Wade JB, Gibson DP, Saeian K, Wegelin JA, et al. Rifaximin improves driving simulator performance in a randomized trial of patients with minimal hepatic encephalopathy. Gastroenterology. 2011;140(2):478–87 e1. doi: 10.1053/j.gastro.2010.08.061 20849805 PMC3020996

[7] Campagna F, Montagnese S, Ridola L, Senzolo M, Schiff S, De Rui M, et al. The animal naming test: An easy tool for the assessment of hepatic encephalopathy. Hepatology. 2017;66(1):198–208. doi: 10.1002/hep.29146 . 28271528

[8] Kawaguchi T, Konishi M, Kato A, Kato M, Kooka Y, Sawara K, et al. Updating the neuropsychological test system in Japan for the elderly and in a modern touch screen tablet society by resetting the cut-off values. Hepatol Res. 2017;47(12):1335–9. doi: 10.1111/hepr.12864 . 28066966

[9] Bass NM, Mullen KD, Sanyal A, Poordad F, Neff G, Leevy CB, et al. Rifaximin treatment in hepatic encephalopathy. N Engl J Med. 2010;362(12):1071–81. Epub 2010/03/26. doi: 10.1056/NEJMoa0907893 . 20335583

[10] Mas A, Rodés J, Sunyer L, Rodrigo L, Planas R, Vargas V, et al. Comparison of rifaximin and lactitol in the treatment of acute hepatic encephalopathy: results of a randomized, double-blind, double-dummy, controlled clinical trial. J Hepatol. 2003;38(1):51–8. Epub 2002/12/14. doi: 10.1016/s0168-8278(02)00350-1 . 12480560

[11] Suzuki K, Endo R, Takikawa Y, Moriyasu F, Aoyagi Y, Moriwaki H, et al. Efficacy and safety of rifaximin in Japanese patients with hepatic encephalopathy: A phase II/III, multicenter, randomized, evaluator-blinded, active-controlled trial and a phase III, multicenter, open trial. Hepatol Res. 2018;48(6):411–23. doi: 10.1111/hepr.13045 29235218

[12] Ohara M, Ogawa K, Suda G, Kimura M, Maehara O, Shimazaki T, et al. L-Carnitine suppresses loss of skeletal muscle mass in patients with liver cirrhosis. Hepatol Commun. 2018;2(8):906–18. doi: 10.1002/hep4.1207 30094402 PMC6078216

[13] Nishikawa H, Shiraki M, Hiramatsu A, Moriya K, Hino K, Nishiguchi S. Japan Society of Hepatology guidelines for sarcopenia in liver disease (1st edition): Recommendation from the working group for creation of sarcopenia assessment criteria. Hepatol Res. 2016;46(10):951–63. doi: 10.1111/hepr.12774 . 27481650

[14] Hanai T, Shiraki M, Watanabe S, Imai K, Suetsugu A, Takai K, et al. Prognostic significance of minimal hepatic encephalopathy in patients with liver cirrhosis in Japan: A propensity score-matching analysis. J Gastroenterol Hepatol. 2019;34(10):1809–16. doi: 10.1111/jgh.14635 . 30779213

[15] Kanda Y. Investigation of the freely available easy-to-use software ’EZR’ for medical statistics. Bone Marrow Transplant. 2013;48(3):452–8. Epub 2012/12/05. doi: 10.1038/bmt.2012.244 23208313 PMC3590441

[16] Mazzaferro V, Regalia E, Doci R, Andreola S, Pulvirenti A, Bozzetti F, et al. Liver transplantation for the treatment of small hepatocellular carcinomas in patients with cirrhosis. N Engl J Med. 1996;334(11):693–9. Epub 1996/03/14. doi: 10.1056/NEJM199603143341104 . 8594428

[17] Kang SH, Lee YB, Lee JH, Nam JY, Chang Y, Cho H, et al. Rifaximin treatment is associated with reduced risk of cirrhotic complications and prolonged overall survival in patients experiencing hepatic encephalopathy. Aliment Pharmacol Ther. 2017;46(9):845–55. doi: 10.1111/apt.14275 . 28836723

[18] Suzuki H, Sezaki H, Suzuki F, Kasuya K, Sano T, Fujiyama S, et al. Real-world effects of long-term rifaximin treatment for Japanese patients with hepatic encephalopathy. Hepatol Res. 2019;49(12):1406–13. doi: 10.1111/hepr.13415 . 31347756

[19] Kawaratani H, Kondo Y, Tatsumi R, Kawabe N, Tanabe N, Sakamaki A, et al. Long-term efficacy and safety of rifaximin in Japanese patients with hepatic encephalopathy: A multicenter retrospective study. J Clin Med. 2022;11(6). doi: 10.3390/jcm11061571 35329897 PMC8948903

[20] Groeneweg M, Moerland W, Quero JC, Hop WC, Krabbe PF, Schalm SW. Screening of subclinical hepatic encephalopathy. J Hepatol. 2000;32(5):748–53. Epub 2000/06/14. doi: 10.1016/s0168-8278(00)80243-3 . 10845661

[21] Schomerus H, Hamster W. Quality of life in cirrhotics with minimal hepatic encephalopathy. Metab Brain Dis. 2001;16(1–2):37–41. Epub 2001/12/01. doi: 10.1023/a:1011610427843 . 11726087

[22] Bajaj JS, Hafeezullah M, Hoffmann RG, Saeian K. Minimal hepatic encephalopathy: a vehicle for accidents and traffic violations. Am J Gastroenterol. 2007;102(9):1903–9. Epub 2007/07/21. doi: 10.1111/j.1572-0241.2007.01424.x . 17640323

[23] Singh J, Sharma BC, Puri V, Sachdeva S, Srivastava S. Sleep disturbances in patients of liver cirrhosis with minimal hepatic encephalopathy before and after lactulose therapy. Metab Brain Dis. 2017;32(2):595–605. Epub 2017/01/11. doi: 10.1007/s11011-016-9944-5 . 28070704

[24] Labenz C, Toenges G, Huber Y, Nagel M, Marquardt JU, Schattenberg JM, et al. Development and validation of a prognostic score to predict covert hepatic encephalopathy in patients with cirrhosis. Am J Gastroenterol. 2019;114(5):764–70. doi: 10.14309/ajg.0000000000000121 . 30848730

[25] Li YY, Nie YQ, Sha WH, Zeng Z, Yang FY, Ping L, et al. Prevalence of subclinical hepatic encephalopathy in cirrhotic patients in China. World J Gastroenterol. 2004;10(16):2397–401. Epub 2004/07/31. doi: 10.3748/wjg.v10.i16.2397 15285027 PMC4576296

[26] Nardone R, Taylor AC, Holler Y, Brigo F, Lochner P, Trinka E. Minimal hepatic encephalopathy: A review. Neurosci Res. 2016;111:1–12. doi: 10.1016/j.neures.2016.04.009 . 27153746

[27] Sharma K. Minimal hepatic encephalopathy diagnostic dilemma with insights regarding its management and impact on quality of life. J Liver Res Disord Ther. 2018;4(4):136–2. doi: 10.15406/jlrdt.2018.04.00116

[28] Sharma P, Sharma BC. Predictors of minimal hepatic encephalopathy in patients with cirrhosis. Saudi J Gastroenterol. 2010;16(3):181–7. Epub 2010/07/10. doi: 10.4103/1319-3767.65189 20616413 PMC3003225

[29] Wang AJ, Peng AP, Li BM, Gan N, Pei L, Zheng XL, et al. Natural history of covert hepatic encephalopathy: An observational study of 366 cirrhotic patients. World J Gastroenterol. 2017;23(34):6321–9. Epub 2017/10/05. doi: 10.3748/wjg.v23.i34.6321 28974899 PMC5603499

[30] Wang JY, Zhang NP, Chi BR, Mi YQ, Meng LN, Liu YD, et al. Prevalence of minimal hepatic encephalopathy and quality of life evaluations in hospitalized cirrhotic patients in China. World J Gastroenterol. 2013;19(30):4984–91. doi: 10.3748/wjg.v19.i30.4984 23946605 PMC3740430

[31] Iwasa M, Sugimoto R, Mifuji-Moroka R, Hara N, Yoshikawa K, Tanaka H, et al. Factors contributing to the development of overt encephalopathy in liver cirrhosis patients. Metab Brain Dis. 2016;31(5):1151–6. doi: 10.1007/s11011-016-9862-6 . 27353278

[32] Miwa T, Hanai T, Toshihide M, Ogiso Y, Imai K, Suetsugu A, et al. Zinc deficiency predicts overt hepatic encephalopathy and mortality in liver cirrhosis patients with minimal hepatic encephalopathy. Hepatol Res. 2021;51(6):662–73. doi: 10.1111/hepr.13601 . 33242359

[33] Sidhu SS, Goyal O, Mishra BP, Sood A, Chhina RS, Soni RK. Rifaximin improves psychometric performance and health-related quality of life in patients with minimal hepatic encephalopathy (the RIME Trial). Am J Gastroenterol. 2011;106(2):307–16. Epub 2010/12/16. doi: 10.1038/ajg.2010.455 . 21157444

[34] Sidhu SS, Goyal O, Parker RA, Kishore H, Sood A. Rifaximin vs. lactulose in treatment of minimal hepatic encephalopathy. Liver Int. 2016;36(3):378–85. Epub 2015/07/24. doi: 10.1111/liv.12921 . 26201713

[35] Pawar VB, Surude RG, Sonthalia N, Zanwar V, Jain S, Contractor Q, et al. Minimal hepatic encephalopathy in Indians: Psychometric hepatic encephalopathy score and inhibitory control test for diagnosis and rifaximin or lactulose for its reversal. J Clin Transl Hepatol. 2019;7(4):304–12. doi: 10.14218/JCTH.2017.00037 31915599 PMC6943207

[36] Chang C, Huang CH, Tseng HJ, Yang FC, Chien RN. Real-world experience of the one-year efficacy of rifaximin add-on to lactulose is superior to lactulose alone in patients with cirrhosis complicated with recurrent hepatic encephalopathy in Taiwan. J Pers Med. 2021;11(6). doi: 10.3390/jpm11060478 34071787 PMC8226737

[37] Kawaguchi T, Suzuki F, Imamura M, Murashima N, Yanase M, Mine T, et al. Rifaximin-altered gut microbiota components associated with liver/neuropsychological functions in patients with hepatic encephalopathy: An exploratory data analysis of phase II/III clinical trials. Hepatol Res. 2019;49(4):404–18. doi: 10.1111/hepr.13300 30589492 PMC6849579

[38] Rodrigo R, Cauli O, Gomez-Pinedo U, Agusti A, Hernandez-Rabaza V, Garcia-Verdugo JM, et al. Hyperammonemia induces neuroinflammation that contributes to cognitive impairment in rats with hepatic encephalopathy. Gastroenterology. 2010;139(2):675–84. doi: 10.1053/j.gastro.2010.03.040 . 20303348

[39] Balzano T, Dadsetan S, Forteza J, Cabrera-Pastor A, Taoro-Gonzalez L, Malaguarnera M, et al. Chronic hyperammonemia induces peripheral inflammation that leads to cognitive impairment in rats: Reversed by anti-TNF-alpha treatment. J Hepatol. 2020;73(3):582–92. doi: 10.1016/j.jhep.2019.01.008 . 30654069

[40] Wijdicks EF. Hepatic encephalopathy. N Engl J Med. 2016;375(17):1660–70. doi: 10.1056/NEJMra1600561 . 27783916

[41] Ong JP, Aggarwal A, Krieger D, Easley KA, Karafa MT, Van Lente F, et al. Correlation between ammonia levels and the severity of hepatic encephalopathy. Am J Med. 2003;114(3):188–93. doi: 10.1016/s0002-9343(02)01477-8 12637132

[42] Ochoa-Sanchez R, Rose CF. Pathogenesis of hepatic encephalopathy in chronic liver Disease. J Clin Exp Hepatol. 2018;8(3):262–71. doi: 10.1016/j.jceh.2018.08.001 30302043 PMC6175755

